# Smartphone-based Video of *Demodex folliculorum* In Biopsied Human Eyelash Follicles

**Published:** 2015

**Authors:** Mithaq Vahedi, Gavin Davis, Michael James Coleman, Brian Steven Garrett, Allen Omid Eghrari

**Affiliations:** 1 Department of Ophthalmology, Johns Hopkins University School of Medicine, Baltimore, Maryland, USA

**Keywords:** Biopsy, Blepharitis, *Demodex folliculorum*, Smartphone, Video

## Abstract

The ability of smartphone technology to document static microscopy images has been well documented and is gaining widespread use in ophthalmology, where slit-lamp biomicroscopy is frequently utilized. However, little has been described regarding the use of smartphone technology to relay video of tissue microscopy results to patients, particularly when a tissue sample integrates motility of organisms as a characteristic feature of the disease. Here, we describe the method to use smartphone video to document motility of *Demodex folliculorum* in human eyelashes, individual results of which can be shown to patients for education and counseling purposes. The use of smartphone video in documenting the motility of organisms may prove to be beneficial in a variety of medical fields; producers of electronic medical records, therefore, may find it helpful to integrate video drop box tools.

## INTRODUCTION

In the one thousand years since Abu al-Qasim of Andalusia first described thyroid biopsy by needle aspiration, physicians across various fields of medicine have utilized biopsy or sampling of tissue to guide diagnosis and management of countless diseases. Whereas most cases involving surgical biopsy are sent to pathologists and information or static images relayed back to the clinician, some tissue and fluid samples associated with infection may benefit from in-office microscopy, to provide rapid diagnostic answers to the patient. For instance, gram stains and wet mounts can be conducted in real time during patient visits and information relayed to the patient immediately.In one survey, over 90% of family physicians reported the presence of microscopy devices in the office (1). Here, we describe the use of smartphone video technology to integrate these two processes: documentation of tissue sample appearance under the microscope and patient counseling in real time during clinical visits.

In contrast to static images, the availability of portable, high-quality video offered by smartphones provides the opportunity to integrate biopsy results immediately into discussion of management, and offers a salient visual image with which patients may engage in discussion. With increasing use of electronic medical records, these may be stored in a database to be associated with patient files.

**Figure 1:**
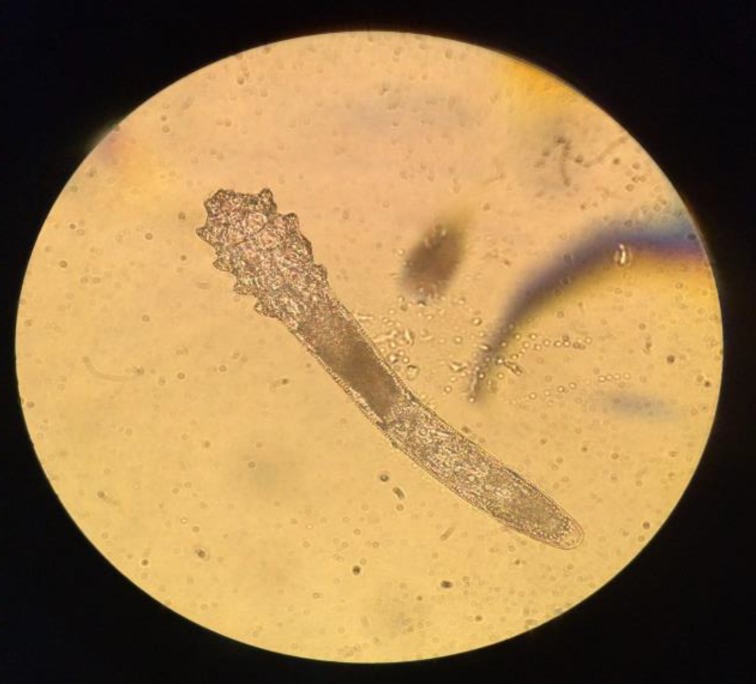
Demodex organism as documented in a static image through a light microscope and iPhone 5S. The chitinous exoskeleton of the organism and spiny edges, as well as recently digested contents, are clearly visible. This particular organism was flushed off the eyelash upon application of the glass slide and was free-floating

When patients present with blepharitis and cylindrical sleeves on the eyelashes, a classic sign of *Demodex folliculorum* infestation, we often conduct epilation of the lash with follicle and record images of the organism using a smartphone and microscope in clinic. These pictures (Fig. 1) are then shown to patients to promote discussion of appropriate lid hygiene.

## METHODS

**Figure 2:**
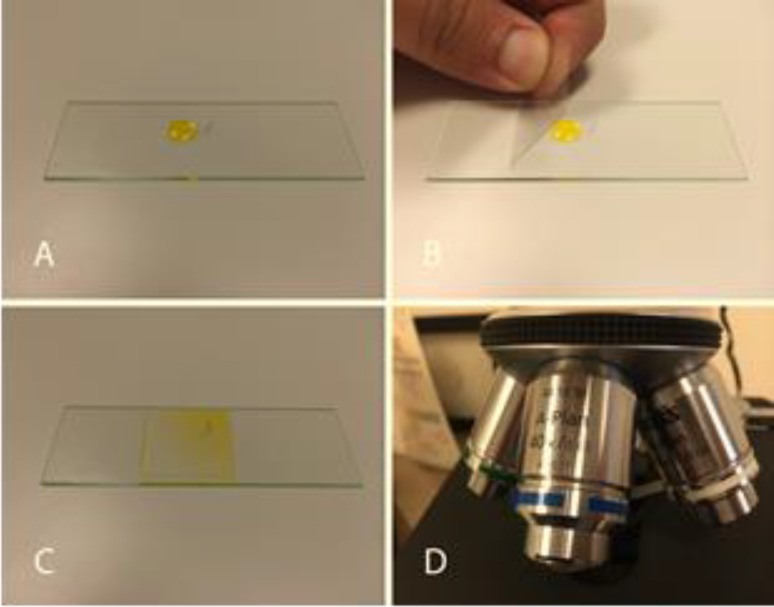
Method of examining Demodex organism on eyelash. A: An eyelash is placed in the center of a glass slide and a small drop of fluorescein placed adjacent to it. B: A glass cover slip is then gently placed on top of the two, with care taken not to flush away organisms with a rapid rush of the drop as it evenly spreads under the cover slip. This may be performed from either direction and may moreover be lowered from the side of the eyelash so that any organisms flushed from the eyelash will still remain under the cover slide. C: The fluorescein drop is evenly spread underneath the coverslip. D: A light microscope is dialed into 40x, which allows adequate visualization of the organism. By holding a smartphone to the eyepiece of the microscope, the organism, and its movement can be recorded

The process of biopsy and imaging is seen in Fig. 2. In each case, an eyelash is removed using fine Jeweler’s forceps at the slit-lamp biomicroscope and placed immediately onto the center of a glass slide. A single drop of fluorescein sodium is placed 4mm away from the lash. A glass cover slip is then gently lowered until the fluorescein slowly and evenly spreads a thin layer of dye onto and around the lash. Placing the slide down first from the side of the lash has the advantage of trapping organisms that may be washed off the lash as the slide is lowered.

Slides are taken with a microscope using 40x power. Video 1 (http://youtu.be/jjjKhl6n6Oc) was captured using an Apple iPhone 5S directly through the optic. We have found it helpful to make the most of the iPhone to visualize better the Demodex organism employing its digital zoom function. Using the native camera software of the iPhone iOS, the Demodex organism, and its movement patterns can be easily observed. Video documentation was performed on the eyelashes of a 30-year-old male with chronic blepharitis. Slit-lamp exam showed typical cylindrical sleeves at the base of the eyelashes. The patient provided informed consent to proceed with epilation and microscopic analysis of eyelashes. Study approval of this report was waived by the Institutional Research Board of the Johns Hopkins University School of Medicine.

Blepharitis is a chronic disease which requires patient motivation and dedication for the best possible treatment outcome; the video of the organisms was shown to the patient, who expressed his motivation to pursue proper lid hygiene using lid scrubs and tea tree products.

## DISCUSSION

Blepharitis is well-known for a frequently chronic course and adherence to a regimen of lid care may be challenging, particularly in the setting of Demodex infestation.(2) *Demodex folliculorum* is a frequent colonizer of eyelash follicles and may contribute to trichiasis or madarosis. (3) Untreated, its chitinous exoskeleton may induce a granulomatous reaction and contribute to chalazion formation.(4) Here, we describe the use of smartphone-based video to document evidence that the organism is present in an eyelash follicle and relay video evidence to the patient instantly, a mechanism that we have found serves as an efficacious motivational tool. We have found that many patients express surprise at the ability to see the organism implicated in their symptoms.

Treatment involves lid hygiene with scrubs to reduce collarettes, and regular application of tea tree oil beyond the 14-day life cycle of organisms to eradicate or reduce pathogenic load,(3) a task for which treatment compliance is essential. Alternative methods of treatment include ivermectin (5); however, systemic application, although less based on compliance, carries systemic risks.

Application of smartphone video technology may prove useful for documentation of a variety of organisms for which movement represents a key feature. Beyond ocular infection, Trichomonas vaginalis is frequently identified by a characteristic pattern of movement appreciated on wet mount, first described in 1836.(6) Associated with the most common non-viral sexually transmitted infection in the world, this organism causes 248 million new cases of infection each year,(7) more than Chlamydia, gonorrhea, and syphilis infections combined.(8) Such high incidence suggests that microscopic smartphone video may find utility on a broad scale. In the clinical setting, smartphone video capture of microscope findings relies on adequate preparation of slides. We have found that Demodex organisms may be easily swept from lashes if a slide cover is quickly or vigorously applied. With Trichomonas, motility is temperature-dependent, with organisms remaining alive at room temperature in phosphate-buffered saline for at least six hours; however, movement may decrease over time.(9) While dedicated video capture devices have been produced for microscopes, we have found that the flexibility and availability of mobile devices offers a unique opportunity to capture findings during patient visits and demonstrate results to patients in real time. Devices used must adhere to patient privacy regulations and should not be mixed with those of personal use.

## DISCLOSURE

The authors report no conflicts of interest in this work.
